# Deep learning-based material decomposition of iodine and calcium in mobile photon counting detector CT

**DOI:** 10.1371/journal.pone.0306627

**Published:** 2024-07-26

**Authors:** Kwanhee Han, Chang Ho Ryu, Chang-Lae Lee, Tae Hee Han

**Affiliations:** 1 Health & Medical Equipment Business Unit, Samsung Electronics, Suwon-si, Gyeonggi-do, Korea; 2 Department of Digital Media and Communications Engineering, Sungkyunkwan University, Suwon-si, Gyeonggi-do, Korea; 3 Department of Artificial Intelligence, Sungkyunkwan University, Suwon-si, Gyeonggi-do, Korea; 4 Department of Semiconductor Systems Engineering, Sungkyunkwan University, Suwon-si, Gyeonggi-do, Korea; Chung-Ang University Gwangmyeong Hospital, REPUBLIC OF KOREA

## Abstract

Photon-counting detector (PCD)-based computed tomography (CT) offers several advantages over conventional energy-integrating detector-based CT. Among them, the ability to discriminate energy exhibits significant potential for clinical applications because it provides material-specific information. That is, material decomposition (MD) can be achieved through energy discrimination. In this study, deep learning-based material decomposition was performed using live animal data. We propose MD-Unet, which is a deep learning strategy for material decomposition based on an Unet architecture trained with data from three energy bins. To mitigate the data insufficiency, we developed a pretrained model incorporating various simulation data forms and augmentation strategies. Incorporating these approaches into model training results in enhanced precision in material decomposition, thereby enabling the identification of distinct materials at individual pixel locations. The trained network was applied to the acquired animal data to evaluate material decomposition results. Compared with conventional methods, the newly generated MD-Unet demonstrated more accurate material decomposition imaging. Moreover, the network demonstrated an improved material decomposition ability and significantly reduced noise. In addition, they can potentially offer an enhancement level similar to that of a typical contrast agent. This implies that it can acquire images of the same quality with fewer contrast agents administered to patients, thereby demonstrating its significant clinical value.

## Introduction

Photon counting detector (PCD)-based computed tomography (CT) is a next-generation CT detector technology that has developed significantly in the past decade. Conventional energy-integrating detectors (EIDs) require a scintillator to convert high-energy X-ray photons into visible light and then detect the converted light in an integrated form. Therefore, the energy information of the individual photons is lost. However, PCD can directly detect X-ray photons without using a scintillator and preserve their energy information [1]. PCD offers several advantages over conventional EID detectors, including reduced electronic noise, increased image resolution, improved contrast-to-noise ratio (CNR), and multiple energy data [2, 3].

One of the main objectives of PCD development is to perform accurate material decomposition via multiple energy data acquisition. Material decomposition methods using dual-energy CT data present the following major disadvantages:

- Inadequate energy separation owing to energy overlap reduces material decomposition accuracy [4].
- Increasing the number of distinct energies and accurately separating them with temporal and spatial alignments is challenging [5].

By contrast, multiple-energy data acquisition using a PCD allows for accurate differentiation of the energy characteristics of each material. Consequently, the clinical potential of PCDs is extremely high because they can solve the problems associated with dual-energy CT. Material decomposition using multiple energy data reduces the use of contrast agents, which increases the accuracy of evaluating internal tissues in the body and facilitates lesion diagnosis in diverse applications such as kidney stone and brain hemorrhage detection [6–8]. Material decomposition has been investigated in various systems.

Conventional material decomposition methods exploit the statistical characteristics of material maps, normalization strategies, and spectral information [9]. However, these methods are restricted by systematic limitations, resulting in increased image noise and inaccurate material decomposition. Various methods have been developed to address these issues, such as material decomposition using iterative reconstruction [10–12] and numerous material maps, including multi-material decomposition (MMD) [13], to improve material decomposition accuracy.

Nevertheless, there are disadvantages to relying on scanner energy reactions and normalization strategies, which involve computationally expensive repetition characteristics and prior knowledge. In particular, a method that relies on prior knowledge can reduce the material decomposition accuracy owing to pulse pileup and charge sharing in PCD [14].

Recently, deep learning has demonstrated remarkable results in material decomposition [15–17]. Among the deep learning techniques, convolutional neural networks (CNNs) [18] have demonstrated outstanding performance in various image-processing tasks and have been widely utilized in medical imaging [19, 20]. However, strict and limited anatomical labeling procedures and insufficient data restrict deep learning-based material decomposition. In a previous study [16], material decomposition based on artificial neural networks (ANNs) was conducted using live animal data from mobile PCD-CT with simplified labeling procedures. However, simple networks and inadequate data can lead to imprecise material decomposition. The network training utilizing customized loss based on the MSE [15, 17, 21] was adopted to minimize pixel-level errors with labels during material decomposition. However, the results were not perfect because the values for the materials were similar. To address these problems, some studies have presented research using the maximum likelihood (ML) estimation approach [22] or combining traditional model-based and ML estimation solutions [23]. However, it still produces noisy results and requires considerable computational cost and training data.

Herein, we propose Unet-based material decomposition (MD-Unet) using modified 3D-Unet and transfer learning with simulated images to address the above problems. Unet [24], a CNN-based network with excellent performance using limited amounts of data, has demonstrated high accuracy in semantic segmentation and has been widely applied in CT imaging [15, 19–21]. The mobile PCD-CT device used for data acquisition could obtain data for low-, mid-, and high-energy ranges in a single scan by setting three threshold values. Because the data for each energy range (bin) represent attenuated X-ray photons at the same location, a spatial correlation exists. Therefore, we modified the network to perform three-dimensional (3D) convolution by converting the data from the three energy bins into 3D data. In addition, we adopted a customized loss function based on the cross-entropy tailored to the three energy bins. Subsequently, to overcome the limitations of the anatomical labeling procedure, simulated phantom images were generated using system information to supplement the insufficient data. Furthermore, the accuracy of the simulation data was improved by reducing the error through pre-processing using material concentration information from the phantom (model 1472 Gammex) and simulated images. The network was pre-trained using the simulated image, and transfer learning was performed on real phantom images. Consequently, the network obtained maps of the target materials of interest, including water, iodine, and calcium.

We generated a simulated image using inserts of different concentrations and sizes for data augmentation. Additionally, elastic deformation, random flip, and random rotation were applied to prevent overfitting owing to the simple shape of the phantom. For the real phantom images, random flip and rotation operations were used to improve the learning performance owing to artifacts and noise. Network testing was conducted using animal experimental datasets, specifically the brain CT images of a beagle, to observe the clinical effects of material decomposition. Conventional least-squares-based material decomposition (MD-LS) [25], total-variation regularized material decomposition (MD-TV) [10], MMD, ANN-based methods, and Unet were used for the performance comparison. The CNR, mean intersection over union (mIoU), and mean dice coefficient (mDSC) for the real phantom images were quantitatively evaluated, and the results were visually compared based on experimental animal data.

## Methods

### Data preparation

All data for this study were acquired using a prototype mobile PCD-CT system. The prototype mobile PCD-CT system replaced the existing EID array of a commercial mobile CT system (CereTom, Samsung Neurologica, Danvers, MA, USA) with a prototype PCD. The geometry of the mobile PCD-CT prototype was identical to that of a commercial mobile CT system (CereTom, Samsung Neurologica, Danvers, USA). However, the PCD was designed to obtain multi-energy data. To achieve this, a photon-counting module was created using an integrated circuit (IC) comprising a 1.4-mm-thick cadmium telluride semiconductor detector. Each photon-counting module featured 80 × 48 pixels with each pixel measured 0.23 × 0.19 mm. The system comprises 48 photon-counting modules, resulting in 3840 and 80 pixels in the row and column directions, respectively. The distance from the source to the isocenter was 227.5 mm, and the maximum field of view was 250 mm. The energy range of the mobile PCD-CT was 30–140 keV, and three energy bins (bin 1:30–50 keV; bin 2:50–65 keV; and bin 3:65–140 keV) of the projection data were acquired in a single scan. Healthy male beagle dogs were used in this study and were injected subcutaneously with 0.05 mg/kg of atropine sulfate (Daihan Pharm, Seoul, Korea). Following a 10-minute interval, the dogs were sedated with 2 mg/kg xylazine (Bayer, ON, Canada) administered intramuscularly. Anesthesia was induced 10 minutes later with a slow intravenous injection of 2 mg/kg alfaxalone (Jurox, NSW, Australia) and maintained with 1–2% isoflurane mixed with 1.0 L/min of 100% O_2_ after intubation. Temperature (37–39°C), pulse (80–120 beats/min), sPO_2_ (>95%), and end-tidal CO_2_ (ETCO_2_) (40–50 mmHg) were monitored during the CT scan. A multi-energy CT phantom dataset was utilized to train and test a deep learning network for material decomposition, and an animal experimental dataset was incorporated for network testing. The scan protocol was as follows: axial scan; tube voltage, 140 kV; current range, 1–7 mA; gantry rotation time, 2 s; and 1440 projections per rotation in a 5 × 6 binning mode, which resulted in 16 slice images in a single rotation with a slice thickness of 0.640 mm at the isocenter. Images were reconstructed using a filtered back-projection (FBP) algorithm with a 250 mm field of view. Each energy bin was preprocessed in accordance with the methodology described in a previous study [26].

### MD-Unet design

**Fig 1:**
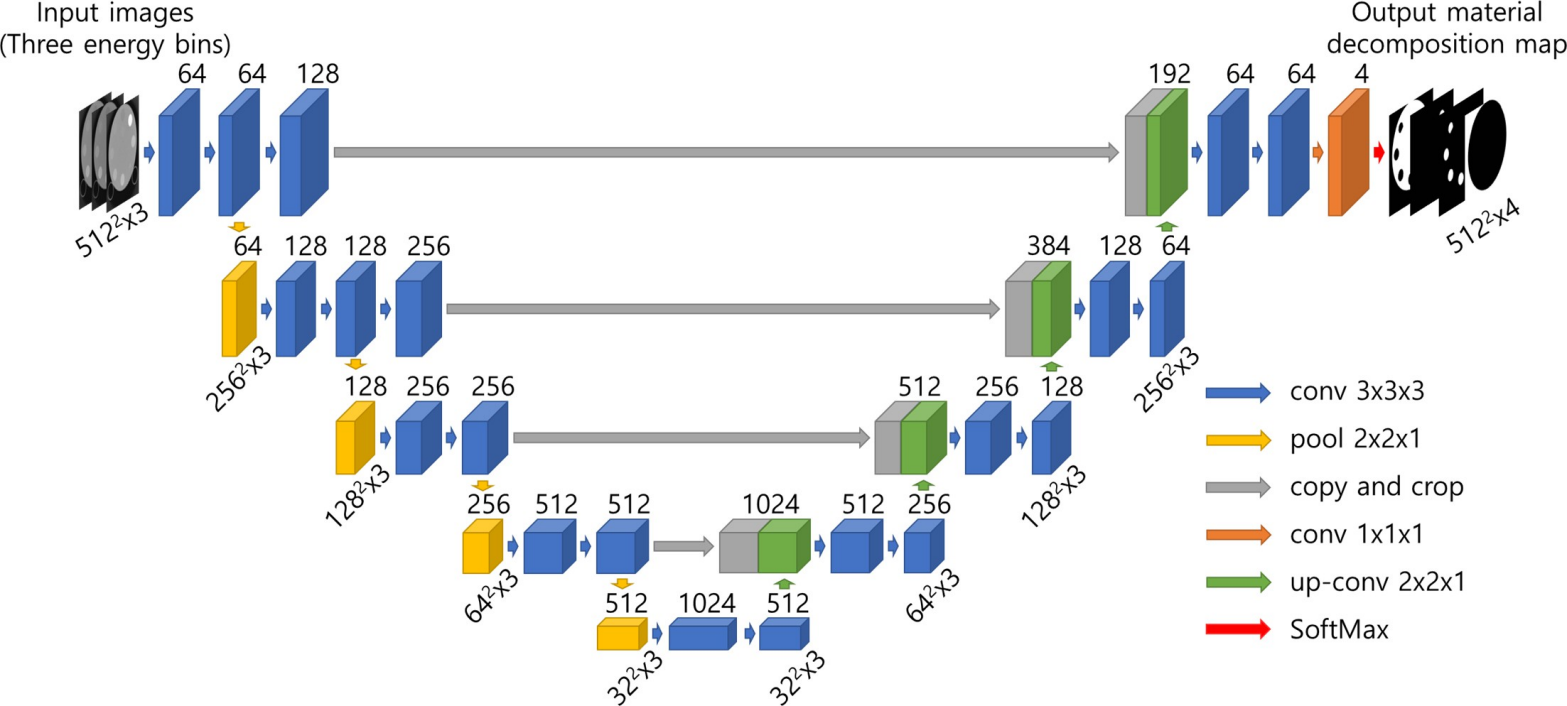
Customized 3D Unet architecture for material decomposition. Each box and arrow represents a feature map and operation, respectively. Resizing operations can be performed on the input data through down-sampling via pooling or up-sampling via up-convolution. The resized feature map is represented in five scales, and the width-height-bin-size of each scale is the same.

To perform material decomposition using deep learning, we selected Unet, a CNN-based network with excellent performance even with limited data. The network was adjusted to fit the system characteristics by modifying it, as shown in Fig 1, and is referred to as MD-Unet. Depending on the threshold settings, the mobile PCD-CT device used for data acquisition collected information across low-, medium-, and high-energy ranges in a single scan. The attenuation of the X-ray photons at the same location correlated with the data from each energy range, demonstrating a local relationship. Thus, we converted the data collected from three different energy ranges (bins) into three-dimensional data for model training.

MD-Unet comprises an encoder and decoder neural architecture, similar to the basic Unet, but with a 3D convolution to reflect the correlation between the bin data. At each layer, 3D convolution, 3D batch normalization, and the ReLU activation functions were executed. 3D max-pooling was applied after every two convolution operations for compression. The number of channels doubled at each scale and increases to 1024 channels at the final scale. The decoder performs the same convolution operation as the encoder. However, instead of compression, up-sampling is achieved via a 3D up-convolution operation after every two convolution operations. Additionally, MD-Unet deploys weighted skip connections on the first and second scales to send information from the corresponding high-resolution layers of the encoder to the decoder, thereby allowing the network to capture finer details in the CT image. The number of channels was specified as the number of materials via a 1 × 1 convolution operation to obtain a map of the materials to be decomposed. In addition, a softmax layer was added to limit the sum of the probability values of all substances at the same position to one. The network can generate substance-specific material maps for water, calcium, iodine, and the background and contains 29 hidden layers. Finally, the customized loss function for training was formulated as follows:

$$Loss=FL\left(f_{GT},f_{P}\right)+\lambda MGE\left(f_{GT},f_{P}\right),$$ (1)

where the focal loss (FL) is the customized focal loss [27] between the MD-Unet output *f*_*p*_ and ground truth *f*_*GT*_. The mean gradient error (MGE) [28] was used to improve edge preservation. FL, which is widely used in deep learning, was leveraged as a customized method to address the problem of class imbalances in cross-entropy loss.


$$FL\left(f_{GT},f_{P}\right)=\frac{1}{N}\sum_{i=Material}-w_{b}{\left(1-f_{Pi}\right)}^{\gamma }f_{GTi}log\left(f_{Pi}\right)$$ (2)

Medical images feature large background areas and non-uniform materials that need to be separated; hence, the materials can significantly affect the total loss and cause instability during early training. To address this issue, we increased the training stability by applying *w*_*b*_ for the background weight and a modulating factor (1-*f*_*Pi*_)^γ^ to the cross-entropy loss, where *γ* = 2 was set empirically and *N* denotes the number of pixels. Additionally, the MGE was used to improve the edge preservation of the material decomposition results.

$$MGE\left(f_{GT},f_{P}\right)=\frac{1}{N}\sum_{i=Material}{\left\{\sqrt{{\left(f_{GTi}*M_{h}\right)}^{2}+{\left(f_{GTi}*M_{v}\right)}^{2}}-\sqrt{{\left(f_{Pi}*M_{h}\right)}^{2}+{\left(f_{Pi}*M_{v}\right)}^{2}}\right\}}^{2}$$ (3)

where *M*_*h*_ and *M*_*v*_ denote horizontal and vertical masks, respectively. The Sobel operator [29], which acts as a high-performance edge detection filter in multiple directions, was used to calculate the pixel-wise gradients in the horizontal and vertical directions. Because material decomposition requires that the boundaries of materials be learned close to *f*_*GT*_, we calculated the pixel-wise gradients. The scaling factor of the MGE, λ, was set to 0.01 based on the experiment.

### Transfer learning using simulation data

#### Simulation image preparation

Although a substantial amount of clinical data is necessary for learning, obtaining ample medical imaging data that reflects the system’s characteristics is challenging. Therefore, we generated simulation data that reflected the system characteristics to augment the training data and conducted transfer learning using only the phantom and simulation data without clinical data. The geometric information of the system and a database of the linear attenuation coefficient (LAC) provided by the National Institute of Standards and Technology were utilized to generate the simulation data. In addition, using the half-value layer measurement values obtained from the system, an X-ray spectrum ranging from 0 to 140 keV was generated using an in-house X-ray simulator. The X-ray spectrum, which reflects the characteristics of the system, was used to generate an element-specific LAC for the target material. Basic FBP algorithms were incorporated to reconstruct the simulated images. Simulation data were generated for three energy ranges: low (bin 1:30–50 keV), medium (bin 2:50–65 keV), and high (bin 3:65–140 keV). An image was created with a structure similar to that of a real multi-energy phantom. Although the concentration trends of the LAC values in the simulated and real images were similar, the LAC values differed owing to the nonlinearity and noise inherent in the system. Therefore, a curve-fitting technique was employed based on the concentration correlation information obtained from real and simulated images. Specifically, the relationship between the concentration and LAC in both types of images was used to minimize discrepancies. Using the following equation, the errors were rectified, and the precision of the simulation outcomes was enhanced:

$$LAC_{real}=a*e^{-b*LAC_{sim}}+c$$ (4)

**Fig 2:**
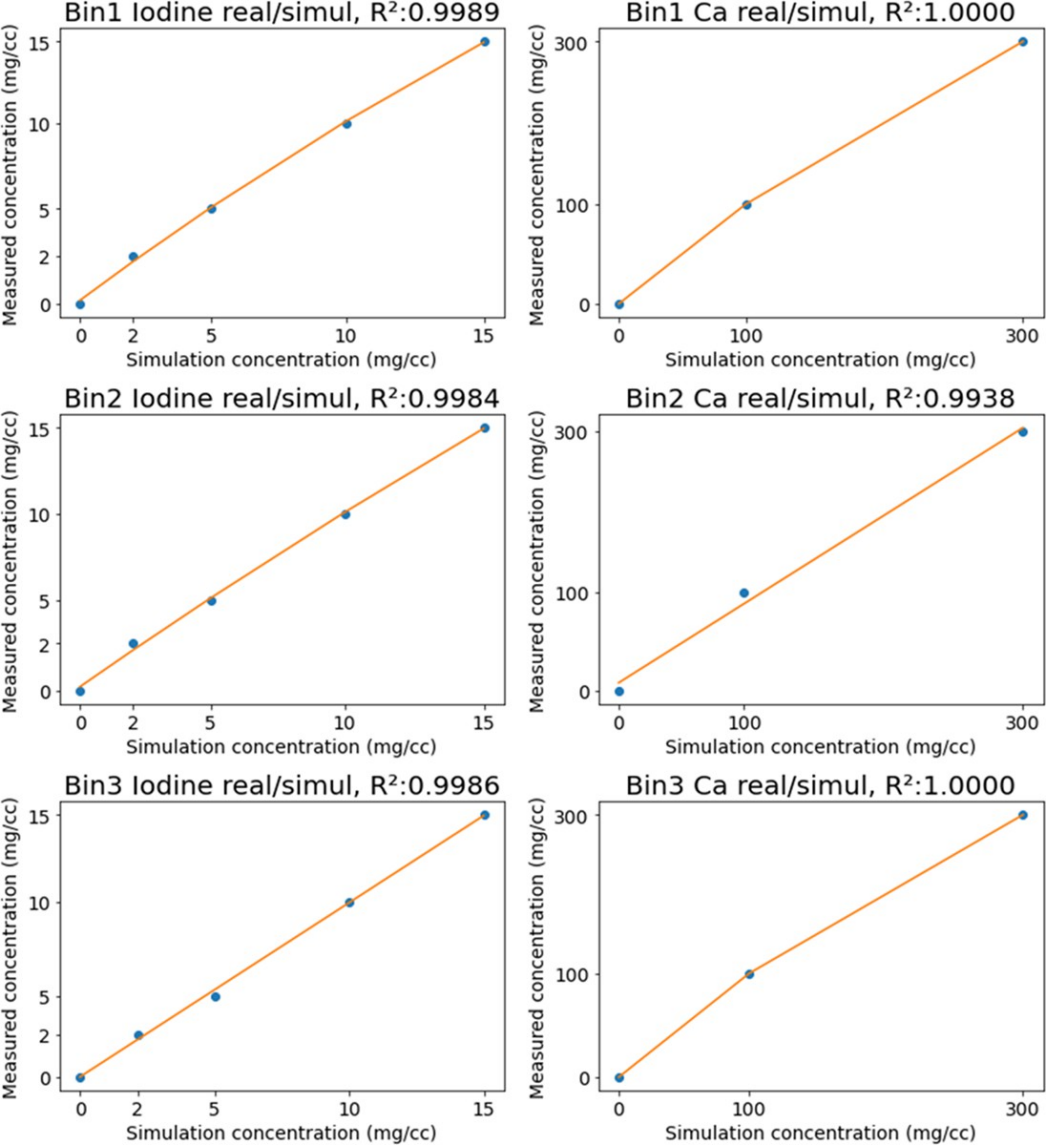
LAC correlation graph based on concentration between real and simulated images. We modeled the LAC correlation for iodine (left column) and calcium (right column). Four types of iodine concentration and two types of calcium concentration information were used. Both materials were measured from concentration-specific inserts of the multi-energy phantom (model 1742 Gammex). The orange line fits the simulation values to the real values; the blue dots indicate pairs of real and simulation data.

We selected a model to accurately estimate the LAC values for simulated and real images, as well as for concentrations that were not present. Parameters a, b, c, and d of the logistic function were determined iteratively using the nonlinear least-squares method. This fitting process reduces the errors in the concentration-dependent LAC correlation between the simulated and real images, as shown in Fig 2. Additionally, a correlation graph with respect to LAC concentration was employed to produce simulated images featuring inserts of diverse concentrations, as shown in Table 1. By employing this model and fitting technique, we improved the simulation accuracy, enabling the effective estimation of LAC values for different concentrations, including those not present in real images. The generated simulation images were utilized for network pre-training before using real data. The weights learned from this training were transferred to the network using real data to improve the learning performance.

**Table 1:** Concentration of real phantom and simulated inserts. Through modeling, we generated six additional calcium-simulated and five iodine-simulated inserts for concentration augmentation. The generated data were used for pre-training.

**Calcium inserts**	**Real concentration**	100, 300 mg/mL
	**Simulated concentration**	100, 200, 300, 400, 500, 600, 900, 1000 mg/mL
**Iodine inserts**	**Real concentration**	2, 5, 10, 15 mg/mL
	**Simulated concentration**	2, 4, 5, 8, 10, 15, 20, 25, 30 mg/mL

#### Transfer learning

**Fig 3:**
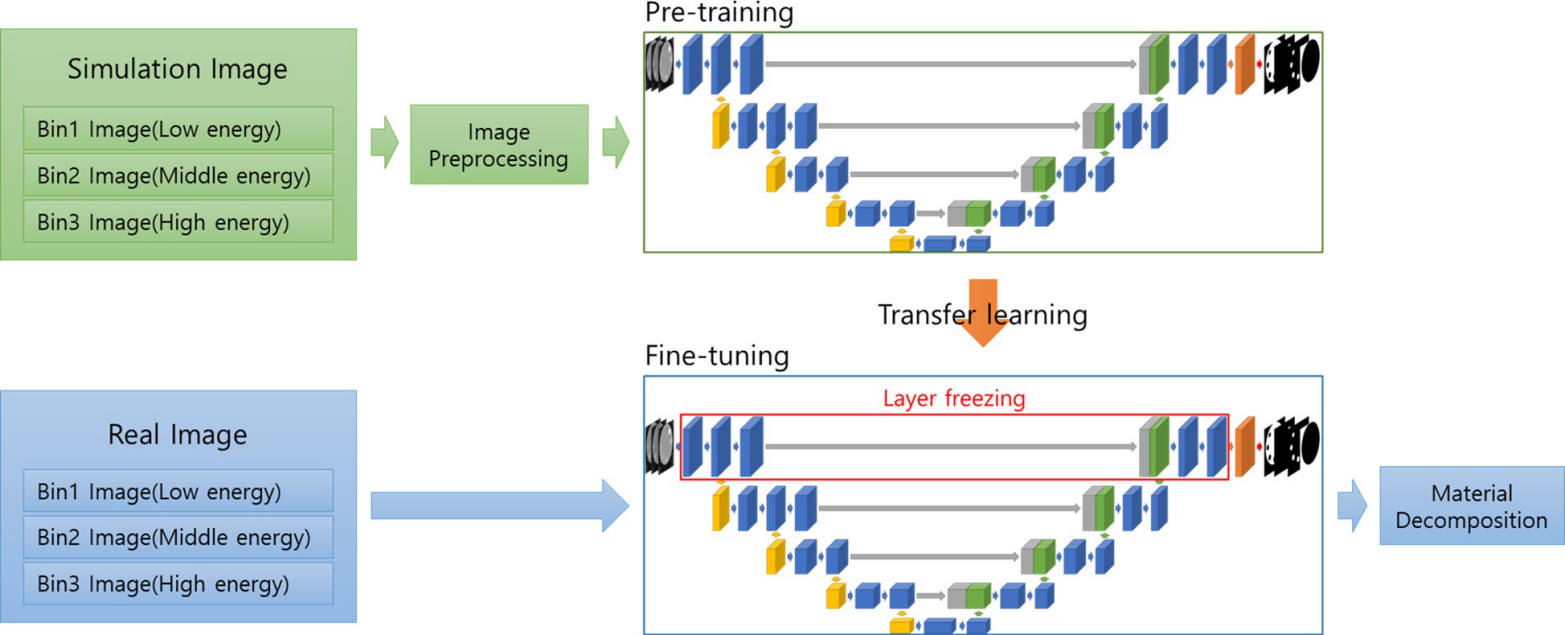
Illustration of transfer learning structure for material decomposition. During pre-training, the entire model is trained using simulated images. Subsequently, during fine-tuning, the first scale layer is frozen, and training and inference are performed using real images.

A schematic representation of the proposed approach is shown in Fig 3. First, three bin images, similar to the real images, were generated via a simulation. The prepared deep-learning network was trained using preprocessed images based on concentration-dependent LAC correlations. For the augmentation strategies, in addition to flipping and rotation, we used elastic deformation and a wide range of simulation phantoms with varying concentrations, sizes, and locations for the inserts to boost the training results. The weights trained using simulation data were transferred to the network and fine-tuned using real data. Moreover, using only real data can cause overfitting of the circular phantom and the loss of elastic deformation features in the trained network. Therefore, the first-scale layer is frozen. Because the real phantom data contained artifacts and noise, only random rotations and flips were performed through augmentation. The final material decomposition maps for water, calcium, and iodine were obtained through training.

### Network training

The epochs for pre-training and fine training were set to 50 and 100, respectively, and the batch size was set to 1. For pre-training, 87 simulated images with three bin images were shuffled and trained, whereas for transfer learning, only 16 image sets with three bin images were shuffled and trained. Consequently, MD-Unet was trained using 103 images.

The Adam optimizer was applied at a learning rate of 0.00001. The network was executed using PyTorch version 1.13 and CUDA 11.7. No additional hyperparameter optimization was performed and a real image set was used as the validation set to monitor overfitting during network training.

### Evaluation and comparison

The results yielded by the conventional and proposed methods were compared by classifying them into two categories: multi-energy phantoms and animal studies. In particular, the results of MD-LS and MD-TV were compared. Additionally, the MMD, which applies volume-preservation principles, was used for comparison. To evaluate the deep learning networks, we compared the results of our network with those of ANNs in previous studies. Additionally, to demonstrate the effectiveness of our network with applied customized loss and transfer learning, we compared it with the traditional Unet. The training of the traditional Unet utilized only cross-entropy loss and showed results from training individually on 16 sets of phantom images without transfer learning. The CNR was employed to evaluate the image quality, and the mDSC, mIoU, and R² score were used to compare the material decomposition accuracy. The CNR was calculated using the average of the region of interest (ROI) for water, calcium, and iodine in each material decomposition result, as well as the average and standard deviation of the background, as follows:

$$CNR=abs\left(\frac{ROI_{AVG}-Background_{AVG}}{Background_{SD}}\right)$$ (5)

The mIoU and DSC are widely used metrics for comparing the performance of segmentation models, particularly in the medical field. In this study, they served as comparison metrics for material decomposition accuracy because the network can obtain decomposition results. The metrics are defined as follows:

$$mIoU\left(f_{gt},f_{p}\right)=\frac{1}{N_{m}}\sum_{i=Material}\frac{\left|f_{GTi}\cap f_{Pi}\right|}{\left|f_{GTi}\cup f_{Pi}\right|}$$ (6)

The mIoU measures the similarity between the predicted results and ground truth labels, where *i* is the material class index and *N*_*m*_ is the number of classes. The mIoU was calculated by obtaining the IoU for each material and averaging the values.


$$mDSC=\frac{1}{N_{m}}\sum_{i=Material}\frac{2|f_{GTi}\cap f_{Pi}|}{\left|f_{GTi}|+|f_{Pi}\right|}$$ (7)

DSC is an effective indicator for evaluating the results of medical image models comprising many small regions because it calculates the harmonic mean of the two regions.

The R² score can be used to evaluate the accuracy of image analysis algorithms. By comparing the actual target material results with the algorithm predictions and calculating R², the performance of the algorithm was assessed. A higher R² score indicates better algorithm performance. This metric is defined as follows.


$$R^{2}score=1-\frac{\sum_{i=Material}{\left(f_{GTi}-f_{Pi}\right)}^{2}}{\sum_{i=Material}{\left(f_{GTi}-\bar{f_{GT}}\right)}^{2}}$$ (8)

We evaluated the material decomposition results using the mIoU, mDSC, and R² score. However, in the case of animals, ground truth is not available. Hence, the CNR was calculated quantitatively, and the accuracy of the material decomposition was assessed visually using the results of previous studies with ANN and conventional Unet as a reference.

### Ethics statement

The authors declare no conflicts of interest. All live animal experiments were performed according to SOP (i.e., SOP-ANI-10(03) for dog management, SOP-ANI-21(01) administration method_dog, SOP-ANI-34(03) environment enrichment, and SOP-IACUC-03(01) post-approval monitoring (PAM) after IACUC approval). In addition, the PAM was conducted to confirm that the experiment adhered to the protocols approved by the IACUC. All procedures used in the animal experiments were approved by the Institutional Animal Care and Use Committee of Daegu–Gyeongbuk Medical Innovation Foundation of the Republic of Korea (permit number: DGMIF-18071004-00). All experiments were performed according to the relevant regulations and ARRIVE guidelines.

## Results

### Phantom studies

**Fig 4:**
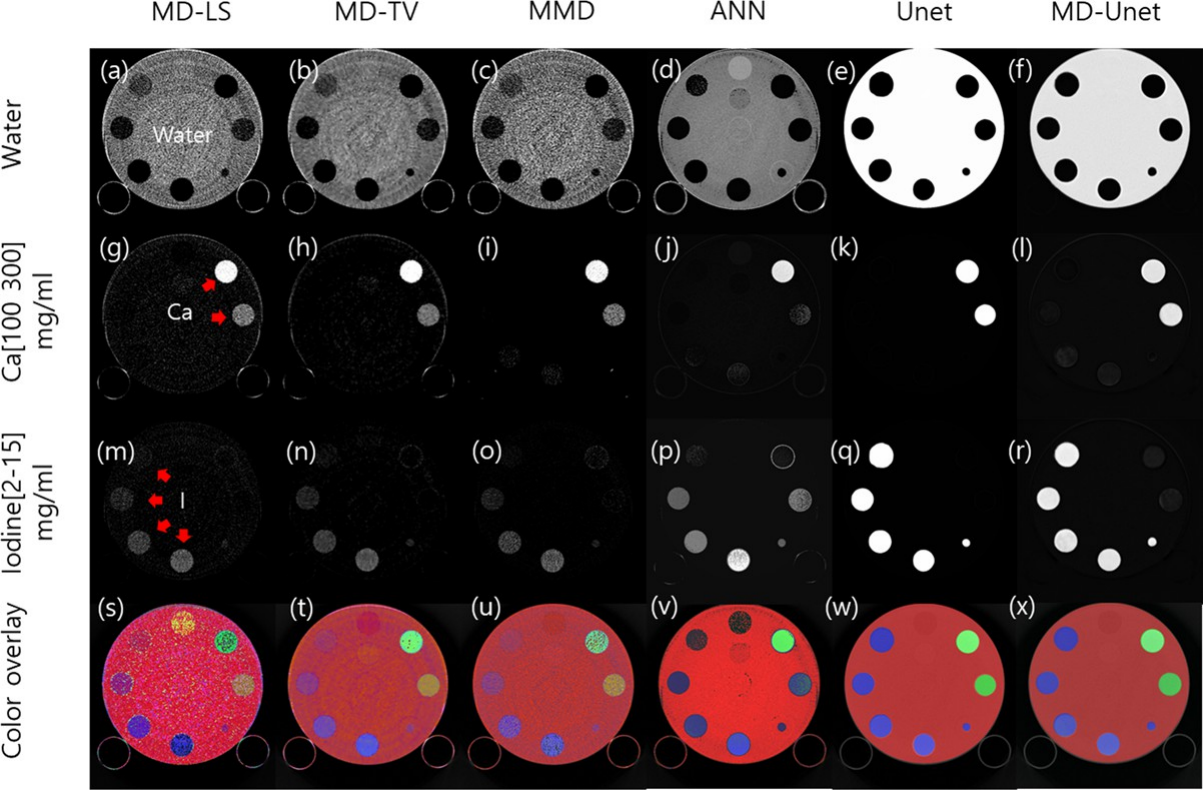
Material decomposition in a multi-energy phantom for each MD-LS, MD-TV, MMD, ANN, Unet, and MD-Unet. The phantom base, fabricated using a solid water material, contained two Ca inserts (30 mm diameter phantom inserts with 100 and 300 mg/mL concentrations) and four I inserts (2, 5, 10, and 15 mg/mL concentrations). The first three rows show the material decomposition maps for each method. The bottom row displays the results of the original image and the material maps overlaid with color.

**Fig 5:**
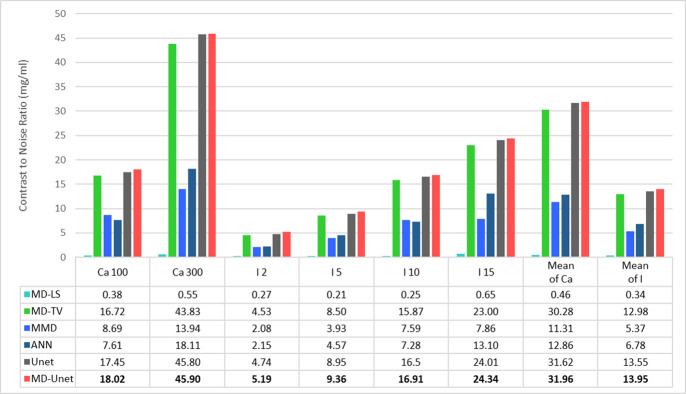
Performance comparison of the proposed MD-Unet and other material decomposition models based on CNR in Eq. (5). CNR was measured using the same ROI per insert in the color overlay images for each model.

Fig 4 shows the material maps and color overlay results of the multi-energy phantom for each material decomposition method. Water, calcium, and iodine were used as the basis materials in all the models. Material decomposition was performed using three energy levels (bins 1, 2, and 3) for each image. Water (a)–(f), calcium (g)–(l), iodine (m)–(r), and color overlay images (s)–(x) were calculated from the phantom data set using the following methods: MD-LS ((a), (g), (m), and (s)); MD-TV ((b), (h), (n), and (t)); MMD ((c), (i), (o), and (u)); ANNs in previous studies ((d), (j), (p), and (v)); conventional Unet ((e), (k), (q), and (w)); MD-Unet ((f), (l), (r), and (x)). The quantitative results for each model are shown in Fig 5 and Table 2. The CNR (mean±SD), mIoU, mDSC, and R² score of the insert were calculated and averaged by concentration levels based on the material decomposition results of each model.

**Table 2:** mIoU, mDSC, and R² score (as expressed in Eqs. (6)-(8), respectively) were calculated using the material decomposition map and ground truth.

Model	mIoU	mDSC	R² score
MD-LS	0.30372	0.29216	0.74109
MD-TV	0.31322	0.30526	0.84631
MMD	0.30689	0.30071	0.82008
ANN	0.30605	0.31460	0.73908
Unet	0.98281	0.98502	0.9539
MD-Unet	0.95758	0.97506	0.98506

### Animal studies

**Fig 6:**
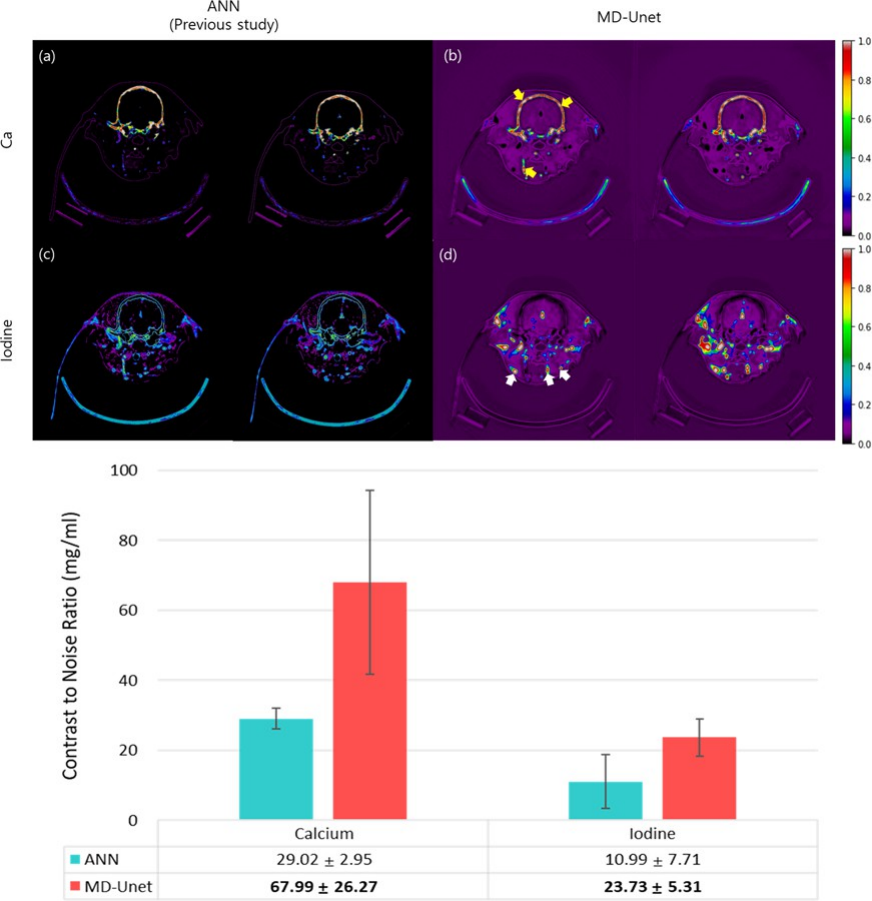
Comparison between previous (left two columns, (a) and (c)) and current studies (right two columns, (b) and (d)) based on a canine’s brain image. The first and second rows represent Ca and I color maps, respectively. CNR (mean±SD) calculated (e) using ROI represented by yellow arrows in (b) and white arrows in (d). The color range for both maps was adjusted so that they ranged from 0 to 1, and a color bar was placed at the far right to illustrate this.

Fig 6 compares the material decomposition results obtained using the previous ANN and MD-Unet. Real animal data were employed for comparison and contrast-enhanced brain images were used to confirm the decomposition abilities of iodine and calcium in each model. The iodine ((a), (b)) and calcium ((c), (d)) levels were calculated from real canine brain images using ANNs (previous studies, (a) and (c) and MD-Unet (b) and (d)). A comparison of the CNR is shown in (e). For comparison, measurements were obtained from the ROI values marked by the arrows in (b) and (d). Arrows closer to red indicate higher concentrations. The color ranges for both were adjusted from 0 to 1.

**Fig 7:**
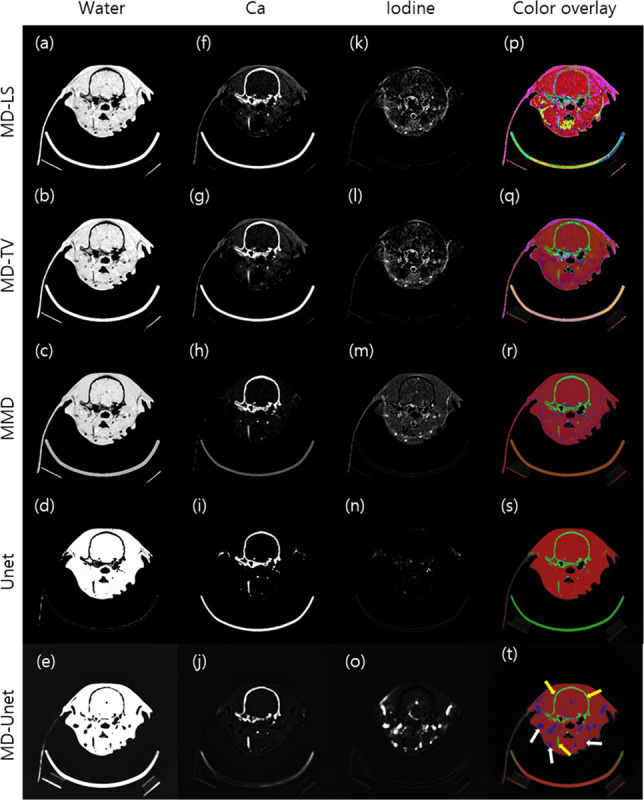
Material decomposition of real animal data for MD-LS, MD-TV, MMD, conventional Unet, and MD-Unet. Spatial distributions of water, I, and Ca for each method are shown in the first, second, and third columns, respectively. The final column represents the results of overlaying the generated material maps from each method as colors onto the original image. The color overlay is used to represent the concentration of each material. The closer the color is to red, the higher the concentration of water. The closer the color is to green, the higher the concentration of Ca. The closer the color is to blue, the higher the concentration of Iodine. (a), (f), and (k) show material maps from MD-LS, (b), (g), and (l) show material maps from MD-TV, (c), (h), and (m) show material maps from MMD, (d), (i), and (n) show material maps from Unet, and (e), (j), and (o) show material maps from MD-Unet. CNR shown in Fig 8 was calculated based on the ROI represented by yellow arrows (Calcium) and white arrows (Iodine) in (t). MD-Unet and Unet models were trained with the same number of epochs and datasets.

**Fig 8:**
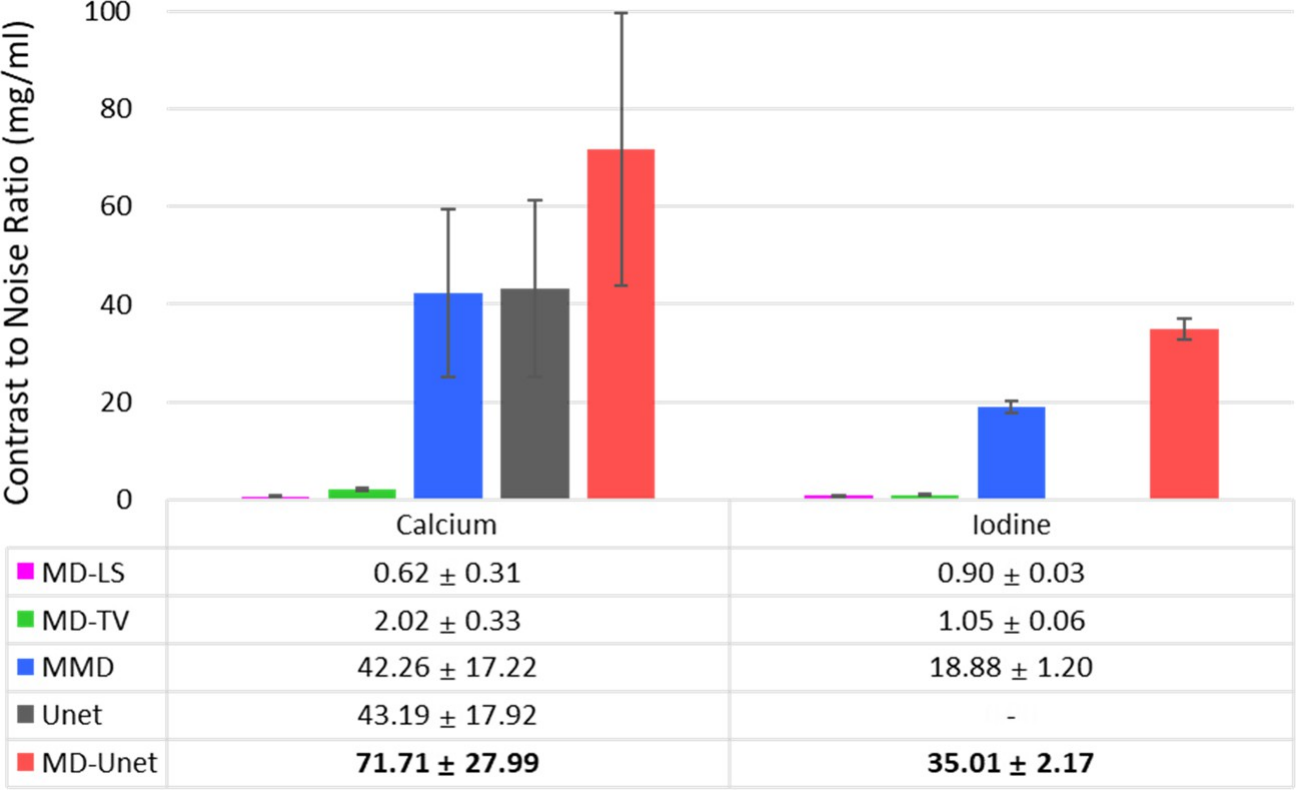
CNR (mean±SD) comparison between the proposed MD-Unet and other material decomposition models in the animal study (Fig 7). CNR was measured using the same ROI (Fig 7T), as shown by arrows in the color overlay images of each model. (–) represents no iodine detected.

Fig 7 shows the material decomposition results for the real animal data obtained using different material decomposition methods, where (a)–(o) show the material maps and (p)–(t) show the original images with color overlays of the material maps. MD-LS, MD-TV, MMD, and conventional Unet were compared with MD-Unet. The ROI markings in (t) were used to facilitate CNR comparison. The yellow and white arrows indicate calcium and iodine, respectively. The CNR measurements for all the methods are shown in Fig 8. The best- performing model is highlighted in bold.

## Discussion

A PCD-CT system acquires multi-energy images in a single scan to execute material and soft tissue decomposition [30]. Based on PCD data obtained from a portable PCD-CT system with a built-in PCD detector, a study of material decomposition based on deep learning was conducted. The MD-Unet network presented enhanced CNRs, Dice scores, and mIOU for material decomposition, surpassing existing methods and demonstrating high accuracy in separating water, calcium, and iodine.

In medical imaging, obtaining accurate results using deep learning is difficult because of a lack of data and difficulties in accurate labeling [31, 32]. The proposed method uses system-specific simulation data for transfer training to solve this problem. Thus, successful material decomposition results were obtained using only 48 phantom images. Fig 4 presents the results obtained using the conventional MD algorithm, ANN, and MD-Unet in the phantom study. MD-Unet generated visually clear and low-noise material decomposition maps compared with conventional methods. This was particularly effective for separating low-concentration iodine inserts (2 mg/mL). However, the differences in concentration in the material map could not be easily confirmed compared to that of conventional methods; nonetheless, this problem can be overcome by performing a color overlay with the original image. Because the network was trained with only a combination of inserts, further validation is necessary for data that include various inserts and their arrangements. Fig 5 and Table 2 present the quantitative evaluation results of MD-Unet. In terms of the CNR, the result was the lowest for MD-LS and the highest for MD-Unet. Similarly, for the mIoU, mDSC, and R² score, MD-LS showed the lowest results. MD-Unet found that the CNR, mIoU, and mDSC improved by 1.7-, 2.0, and 2.1 times on average, respectively, and the R ² score was the highest in MD-Unet, indicating improved noise reduction and decomposition accuracy. However, Unet yielded the best results for the mIoU and mDSC, which is attributable to the overfitting of the Unet network to the shape of Gammex.

For the animal study, we compared the performance of MD-Unet with that of our previous study and a conventional MD algorithm. Fig 6 shows the material decomposition results of each method. Unlike the results reported in previous studies, MD-Unet distinguished between iodine and calcium. Moreover, the bone structure in the neck area (bottom yellow arrow in Fig 6B), which is not visible on the calcium map, was precisely detected. In a quantitative evaluation using CNR, MD-Unet achieved a higher CNR compared to the existing methods, as it separated the target material with higher brightness values and lower background noise. In addition, MD-Unet showed a higher CNR for iodine and calcium than that of the previous method (Fig 6E). Figs 7 and 8 show the material decomposition results of the MD algorithm, Unet, and MD-Unet, respectively. The deep-learning-based material decomposition techniques showed robustness to noise and artifacts, and the proposed MD-Unet performed better at separating iodine and calcium than the conventional Unet (Fig 8). In addition, low-contrast areas were successfully separated. However, despite the augmentation, the separation was not accurate in a specific region learned using only a certain form of the Gammex phantom. A problem occurred when generating the iodine map (Fig 7O), and an error occurred when the ear disappeared from the material map because the ear area was regarded as background in the animal data (Fig 7E). Thus, inadequate data on thin and protruding shapes resulted in inaccurate results. Therefore, additional training data with diverse shapes are required to overcome these limitations. Moreover, due to insufficiently validated clinical datasets, the verification and statistical power of the results were limited. In addition, the results may not fully represent the overall attributes of the method, and their stability may be affected by numerous MD-Unet parameters. Therefore, further clinical studies are warranted.

While the PCD detector in our system can support multiple thresholds, including those specific to K-edges, we limited our analysis to three bins for several reasons. Firstly, generating bin-specific images with clinical efficacy requires each bin’s data to have a sufficient count rate. Employing multiple thresholds can decrease count rates for each bin’s image, compromising image quality and potentially hurting diagnosis. To ensure the clinical utility of the generated images, appropriate count rates, and thresholds are carefully selected settings. Secondly, this study focused on separating clinically significant materials such as iodine, calcium, and water. These materials can be separated using the characteristics of the iodine K-edge (33.2 keV) [16]. This approach allows us to achieve accurate material decomposition while maintaining the diagnostic quality of the images. To optimize the spectral information utilized in our study, we configured the three bins to cover the ranges of 30–50 keV, 50–65 keV, and 65–140 keV, excluding the noise region below 30 keV. This configuration enables us to leverage the spectrum information ranging from 30 to 140 keV, sufficient to distinguish and separate the target materials accurately. We acknowledge that utilizing a more significant number of spectral channels has the potential to enhance material differentiation. However, our approach strikes a balance between leveraging the spectral capabilities of PCD-CT and maintaining practicality in data acquisition, processing, and analysis. Future studies could investigate the impact of increasing the number of channels on material decomposition performance while considering the trade-offs in terms of noise, dose, and computational complexity.

## Conclusions

We evaluated and compared MD-Unet with other material decomposition methods using a mobile PCD-CT prototype. The results demonstrated improved material decomposition accuracy and CNR when using MD-Unet, even with limited real data. In addition, the utility of CT segmentation for brain imaging studies was verified, demonstrating contrast-enhanced CT comparable to that of a standard contrast agent concentration using only a minimal amount of contrast agent.
